# *Solanum tuberosum* and *Lycopersicon esculentum* Leaf Extracts and Single Metabolites Affect Development and Reproduction of *Drosophila melanogaster*

**DOI:** 10.1371/journal.pone.0155958

**Published:** 2016-05-23

**Authors:** Emanuela Ventrella, Zbigniew Adamski, Ewa Chudzińska, Mariola Miądowicz-Kobielska, Paweł Marciniak, Ender Büyükgüzel, Kemal Büyükgüzel, Meltem Erdem, Patrizia Falabella, Laura Scrano, Sabino Aurelio Bufo

**Affiliations:** 1 Department of Sciences, University of Basilicata, Potenza, Italy; 2 Electron and Confocal Microscope Laboratory, Adam Mickiewicz University, Poznań, Poland; 3 Department of Animal Physiology and Developmental Biology, Adam Mickiewicz University, Poznań, Poland; 4 Department of Genetics, Adam Mickiewicz University, Poznań, Poland; 5 Department of Molecular Biology and Genetics, Bülent Ecevit University, Zonguldak, Turkey; 6 Department of Biology, Faculty of Arts and Science, Bülent Ecevit University, Zonguldak, Turkey; 7 Bülent Ecevit University Ahmet Erdoğan Vocational School of Health Services, Zonguldak, Turkey; 8 Department of European and Mediterranean Cultures, University of Basilicata, Matera, Italy; CNRS, FRANCE

## Abstract

Glycoalkaloids are secondary metabolites commonly found in Solanaceae plants. They have anti-bacterial, anti-fungal and insecticidal activities. In the present study we examine the effects of potato and tomato leaf extracts and their main components, the glycoalkaloids α-solanine, α-chaconine and α-tomatine, on development and reproduction of *Drosophila melanogaster* wild-type flies at different stages. Parental generation was exposed to five different concentrations of tested substances. The effects were examined also on the next, non-exposed generation. In the first (exposed) generation, addition of each extract reduced the number of organisms reaching the pupal and imaginal stages. Parent insects exposed to extracts and metabolites individually applied showed faster development. However, the effect was weaker in case of single metabolites than in case of exposure to extracts. An increase of developmental rate was also observed in the next, non-exposed generation. The imagoes of both generations exposed to extracts and pure metabolites showed some anomalies in body size and malformations, such as deformed wings and abdomens, smaller black abdominal zone. Our results further support the current idea that Solanaceae can be an impressive source of molecules, which could efficaciously be used in crop protection, as natural extract or in formulation of single pure metabolites in sustainable agriculture.

## Introduction

Plants produce several kinds of organic compounds known as secondary metabolites [1]. Most of them play important roles in plant protection against pathogens and herbivores [2]. The qualitative—quantitative relationships among these compounds are often species-specific, and they can sometimes be used as important features in plant taxonomy [3]. Secondary metabolites can be classified on the basis of different criteria, such as their chemical structure, composition, solubility in solvents, and/or synthesis pathways [4, 5]. They can be produced in an active state or as prodrugs, which are activated upon wounding, infection or in the body tissues of herbivores [4]. Glycoalkaloids (GAs) are very important secondary metabolites produced by Solanaceae plants as natural defenses against herbivores and phytopathogens. They are present in all parts of the plant in their active form, with the highest concentrations in leaves, unripe fruits and flowers. The structure of these compounds varies. GAs contain nitrogen and are constituted by a glycosidic polar group with three or four monosaccharide units and an aglycone formed by a steroid skeleton and a basic portion [6].

The effects of several GAs on insect physiology have been described in many studies [7–10]. The toxicity induced by GAs can be associated with their membrane-disruptive properties, their inhibition of acetylcholinesterase activity [4] and oxidative stress induction [9]. The composition of the carbohydrate side chain and the nature of the aglycone are fundamental in defining GAs different biological activities that are generally also influenced by their concentration [6].

Increased mortality of the potato leafhopper *Empoasca fabae* has been associated with increased concentration of GAs in the diet; α-tomatine has been proved to cause high acute toxicity [95%] already at concentration of 0.03% on synthetic diet [11]. Similarly, reduced fecundity, decreased feeding and increased mortality were observed for peach potato aphids *Myzus persicae* fed on an artificial diet containing 80-160 mg GA/100 ml of diet [12]. Solamargine, solasonine and α-tomatine inhibited larval growth of the red flour beetle *Tribolium castaneum* and α-tomatine also showed inhibitory activity on tobacco hornworm, *Manduca sexta* [13]. Moreover, potato GAs showed ovicidal effect and repellent activity against *Spodoptera exigua* moths [14] and decreased the frequency of the *Zophobas atratus* heart contractions causing irreversible or fast and reversible cardiac arrests [15, 16]. α-Solanine affected *Galleria mellonella* development, fecundity and fertility, and disturbed prooxidant-antioxidant balance [9]. Similar effects were observed when crude extract from potato leaves was tested on *Galleria mellonella* [10].

The bioactivity exerted by certain secondary metabolites of plants has given rise to the convincement that many of them could be experimented as valid alternative to the use of synthetic pesticides [17]. Several plant alkaloids are traditionally used as insect repellents in developing countries [18, 19] and have stimulated the attention of many researchers [9, 12, 13, 16, 20, 21]. Furthermore, it should be accounted that most of the chemical insecticides possess a broad spectrum of action. Therefore, they may cause significant environmental pollution affecting non-target species and humans as consumers of agricultural products. Frequent and incorrect usage of chemical insecticides causes the increasing of resistant pest strains and reduces the number of molecules available in the effective control of pests. These considerations account for the increased attention to the search for new molecules with insecticidal activity, like botanic or animal substances [22]. Frequently, cellular and molecular activity of natural substances, including GAs, resembles activity of synthetic insecticides [21]. However, introduction of new substances, also of natural origin, into environment always undergoes strict rules. Their lethal and sublethal toxicity should be carefully studied. Also the selectivity and range of toxicity must be checked. Therefore, activity against various species must be tested and, as soon as possible, the mechanism of action should be clarified. GAs toxicity against some model organisms can be compared and integrated among laboratories and research groups considering that for these organisms physiological, biochemical and molecular knowledge are available. Not only mortality, but also sublethal effects are important when toxicity of any substance is described [23, 24]. In the present paper we examine the effects induced by extracts obtained from potato (*Solanum tuberosum*) and tomato (*Lycopersicon esculentum*) leaves and their main secondary metabolites, i.e. the GAs α-solanine, α-chaconine and α-tomatine on development of two consecutive generations of wild-type *Drosophila melanogaster*, to test lethal and sublethal toxicity on insects. This laboratory model organism has a great number of advantages: a high number of offspring, a short life cycle, a simple and cheap breeding and an easy way to distinguish male and female imagoes. Moreover, the knowledge of physiology and biology of this organism allows a deeper understanding about mechanisms of action of analyzed substances.

Fruitflies are commonly used as a model organism for many biological processes including toxicity testing [25–27]. Taking into account the advantages mentioned above, *D*. *melanogaster* seems to be a good model to study compare and integrate toxic effects of xenobiotics. Our aims were [i] to study acute and sublethal activity of GAs against *D*. *melanogaster*, [ii] to evaluate differences between the activity of plant extracts and pure GAs, [iii] to verify if the exposure to tested GAs affected next, non-exposed, generation.

## Materials and Methods

### Insects

The wild-type *Drosophila (Sophophora) melanogaster* Meigen flies (Diptera: Drosophilidae) were obtained from a culture maintained at the Department of Genetics, Adam Mickiewicz University, Poznań, Poland. The experiments were carried out under the laboratory conditions at temperature of 23 ± 2°C and at relative humidity of 60 ± 5%. Virgin females of wild type were collected and crossed to males. After mating overnight, the flies were transferred to vials containing the tested samples and left there for 24 h. Mated flies were then transferred to fresh vials and maintained there for 10 days to monitor the presence of viable offspring.

### Chemicals

Pure α-chaconine (≥ 95%) and α-solanine (≥ 95%) were obtained from LabService Analytica (Italy), α-tomatine was supplied by Sigma-Aldrich (Germany). Commercial α-tomatine contained dehydrotomatine as impurity (α-tomatine: dehydrotomatine 10:1 in weight). Acetic acid, formic acid (99%), LC-MS grade methanol and LC-MS grade acetonitrile were obtained from Carlo Erba (Italy), while propionic acid and ethylic ether were supplied by POCH S.A. (Poland).

### Plant material and sample extraction

Young and small leaves were harvested in May 2011, before fruit appearance from the same stem position [top] of potato plants (*Solanum tuberosum* L.) cultivar Désirée and tomato "cherry" (Lycopersicon esculentum Mill.) grown in a field of Castellana Grotte (40°53′15″ N-17°12′34″ E, Bari, Italy). The plants were grown on private land after owner permission to conduct the study on this area; all the studies did not involve endangered or protected species. The vegetable material was washed and stored at -20°C to arrest maturation. The GAs extraction was performed according to an optimized method [28]. The vegetable samples were lyophilized and ground to a fine powder using a laboratory mill; then, the same procedure of extraction was employed for each freeze-dried sample. In centrifuge tubes 1.5 g of sample were placed in 20 mL of 1% acetic acid aqueous solution. To facilitate contact between plant tissue and extraction solvent, the suspension was stirred for about 2 hours and then centrifuged at 6000 rpm for 30 minutes. The obtained pellet was re-suspended in 5 mL of 1% acetic acid, shaken, centrifuged again and the two supernatants were mixed together. To remove solid particles, each extract was filtered by means of a single-use 0.22 mm nylon filter (Whatman, Maidstone, UK) and then was injected into the Liquid Chromatography/ Mass Spectrometry (LC/MS) system. For determination of extract bioactivity, the liquid phase was eliminated by means of lyophilization.

### Characterization of extracts by LC/ESI-MS

Liquid Chromatography/Electrospray Ionization-Mass Spectrometry (LC/ESI-MS) analysis was carried out in positive mode using a LCQ Classic quadrupole ion trap mass spectrometer QITMS (ThermoFinnigan, San Jose, CA, USA) equipped with a binary pump and a solvent degasser (Spectra System P4000, USA). The column was a Supelcosil LC-ABZ, amide-C16 (5 μm, 250 x 4.6 mm) with a guard column of the same material (Supelco Inc., Bellefonte, PA, USA), which proved to be optimal for GAs chromatographic detection for its high resolution and short analysis time [28]. The used eluents were methanol and 0.1% formic acid in water, while acetonitrile and water lightly acidified with formic acid were used as dilution solvents. Concentrations of main GAs identified in the plant extracts were determined using purchasable pure GA standards.

### Samples of bioactivity assays

The extracts of potato or tomato leaves were lyophilized and re-dissolved in water to obtain the stock solutions of each at concentration 1000 μM of their main component metabolite (α-chaconine and α-tomatine, respectively). Stock solutions at concentration 1000 μM of pure single GAs (α-chaconine, α-solanine and α-tomatine) were prepared by means of their dissolution in 0.1% acetic acid in water (slightly acidic pH values enhanced solubilization of GAs). The assayed solutions were prepared by dilution of the stock solutions to the desired concentrations in water.

### Effect of extracts and pure GAs on development of *D*. *melanogaster*

All stocks were maintained and experiments run on modified sugar/yeast (SY) culture medium (25 g of yeast, 230 g of sugar, 20 g of agar, 100 g of flour, and 8.6 mL of propionic acid in 1 dm3 of distilled water). The tested samples (leaf extracts or single pure GAs) were added to the culture medium which was poured in each vial to obtain a series final concentrations of 0.005, 0.05, 0.5, 5 and 50 μM in the medium. Such a wide range of concentrations was done to observe possible hormetic, sublethal or lethal effects. In order to check the probable effect of solvent which was used to prepare stock solutions of each GA, 0.1% of acetic acid in water was added to culture medium administrated to the control group. Three fertilized females of the same age (7 days) in each vial for 24 hours to lay eggs (approximately 100 eggs per vial). After 24 hours the imagoes were removed from the vials and the development of organisms present in vials, named as parental or P generation, was observed. Numbers of third instar larvae and pupae (visible on surface of substrata and vial’s walls) and imagoes (males and females) were counted throughout the test. The flies were removed from vials after their imaginal molt, sex of imagoes was checked under ethylic ether anaesthetization. The 50% of developmental time (time in days, when 50% of population reached larval, pupal and imaginal developmental stage in vials, T50) was calculated. This factor illustrates the effect of tested substances on the time of development. Percentage of dead pupae and malformed imagoes (i.e. with improperly developed legs or wings, smaller or significantly harmed in the other way) were also determined. Malformed insects were photographed using Stero Lumar V12. Since *D*. *melanogaster* eggs lay in agar-stabilized substratum, the simple method of placing a defined number of eggs on the extract-treated substrata is difficult. The procedure of eggs separation would demand either chemical dissolution of agaric substrata or mechanical separation of eggs, using a stereomicroscope (eggs are smaller than 0.5mm in length). Both methods could destroy eggs. Next, the hatched larvae naturally prefer to feed within the culture medium rather than on it. Hence, it is not possible to check precisely the number of larvae or their developmental stage. The model we propose is less invasive and does not cause additional stress for the larvae.

A factor describing the final number of organisms in comparison to control (FNO) was determined to compare the results:

$$FNO=\frac{T-C}{C}\;100$$

where: T—final number of organisms counted in tested sample-containing vials; C—final number of organisms counted in control vials.

Next, three adult females and three adult males from each parent generation (P) were transferred to another vial containing the control medium vial. After mating overnight, mated flies laid eggs and organisms of first generation (F_1_) completed their development. The experiments were carried out in triplicates along with control sets.

### Statistical analysis

The data analysis was performed through R 2.10.1 software. Results were expressed as mean± SD of three replicates. The mean differences between treatments and control were statistically compared using U-Mann Whitney nonparametric test. Values at p < 0.05 were regarded as significantly different from the control. Correlation between concentration of extracts or pure alkaloids and measured factors (mortality, malformations, FNO, T_50_) was calculated and shown if the value was higher than 0.7 or lower than -0.7.

## Results

The extract of potato leaves contained 4.80 ± 0.32% of α-chaconine and 3.04 ± 0.15% of α-solanine, together with other minor GAs, especially dehydrochaconine and solanidadienol chacotriose, which possess the same glycosidic group of α-chaconine (chacotriose) but different aglycones. Extract of tomato leaves displayed the presence of the major GAs (2.95 ± 0.25%), α-tomatine, and other two minor GAs sharing with it the same glycosidic group, lycotetraose, namely dehydrotomatine and filotomatine.

The tested extracts and single pure metabolites affected the final number of organisms counted and the time of their development. It could be assumed that when insects were exposed to extracts, a smaller number of organisms survived and the sublethal effects were more significant, with respect to their exposition to pure metabolites. The results reported in Table 1 show the effect of different concentrations of *S*. *tuberosum* leaf extract, α-chaconine and α-solanine on parental (exposed) generation of *D*. *melanogaster* in terms of T50 values for larvae, number of final organisms and FNO factor values for imagoes, dead pupae and malformed imagoes percentage. In the majority of cases, non-linear effects were observed. Therefore, very often, correlation coefficient had a weak or moderate value. Only mortality caused by extract and pure α-chaconine, but not α-solanine, correlation was strong or very strong, respectively. The extract and α-chaconine caused significant decrease of developmental time (T_50_). On the other hand, we observed strong negative correlation between α-solanine and time of larval development. However, α-solanine decreased developmental time less, than *S*. *tuberosum* extract and α-chaconine.

**Table 1:** Effect of *S*. *tuberosum* leaf extract, α-chaconine and α-solanine on parental (exposed) generation of *D*. *melanogaster*.

Concentration [μM]	Larvae	Pupae		Imagoes			
	T_50_ [days]	Mortality [%]	T_50_ [days]	Malformations [%]	N° final organisms	FNO	T_50_ [days]
***S*. *tuberosum* extract**							
Control	14.5 ± 0.4	0.00 ± 0.00	21.2 ± 2.3	0.00 ± 0.00	32.67 ± 4.51	0.0	24.2 ± 1.6
0.005	10.0 ± 0.9*	5.56 ± 4.81	11.8 ± 0.4*	15.39 ±3.00	13.67 ± 4.62*	- 58.2	14.9 ± 0.5*
0.05	11.0 ± 1.1*	2.78 ± 2.55	12.0 ± 0.4*	12.54 ± 8.17	19.33 ± 11.15	- 40.8	15.6 ± 0.2*
0.5	9.8 ± 1.0*	0.00 ± 0.00	12.0 ± 1.0*	6.02 ± 3.61	10.33 ± 6.81*	- 68.4	15.4 ± 0.4*
5	8.6 ± 0.6*	2.78 ± 2.55	11.9 ± 0.8*	21.09 ± 5.00*	11.67 ± 2.08*	- 64.3	15.0 ± 0.4*
50	9.7 ± 0.8*	8.33 ± 8.10	11.7 ± 0.6*	83.63 ± 6.76*	19.33 ± 13.05	- 40.8	15.1 ± 0.6*
Correlation coefficient	nc	0.77	nc	0.96	nc	nc	nc
**α-chaconine**							
Control	14.5 ± 0.4	0.00 ± 0.00	21.2 ± 2.3	0.00 ± 0.00	32.67 ± 4.51	0.0	24.2 ± 1.6
0.005	11.9 ± 2.2	0.00 ± 0.00	16.1 ± 3.6*	9.01 ± 6.86	19.33 ± 10.02	- 40.8	20.6 ± 1.4
0.05	11.4 ± 2.5	3.70 ± 3.55	14.9 ± 1.0*	25.15 ± 14.29*	21.00 ± 11.36	- 35.7	18.2 ± 2.6*
0.5	11.6 ± 0.6	5.13 ± 5.02	12.2 ± 0.2*	9.63 ± 4.88	34.00 ± 6.24	+ 4.1	15.8 ± 0.5*
5	11.3 ± 0.9	7.51 ± 4.44	12.5 ± 0.9*	11.91 ± 4.16	32.00 ± 23.30	- 2.1	16.6 ± 1.0*
50	10.5 ± 0.3	17.96 ± 11.32	12.1 ± 0.0*	23.60 ± 10.83*	30.00 ± 8.54	- 8.2	15.8 ± 0.4*
Correlation coefficient	nc	0.93	nc	nc	nc	nc	nc
**α-solanine**							
Control	14.5 ± 0.4	0.00 ± 0.00	21.2 ± 2.3	0.00 ± 0.00	32.67 ± 4.51	0.0	24.2 ± 1.6
0.005	14.0 ± 0.4	4.54 ± 4.22	16.7 ± 1.8	12.73 ± 12.18	29.33 ± 9.07	- 10.2	21.1 ± 2.1
0.05	14.5 ± 0.5	9.57 ± 8.35	20.8 ± 1.0	28.40 ± 9.52*	27.00 ± 5.57	- 17.4	27.1 ± 3.7
0.5	16.2 ± 1.9	0.00 ± 0.00	19.9 ± 2.5	23.58 ± 11.98	29.33 ± 13.05	- 10.2	25.9 ± 3.2
5	15.7 ± 0.3	14.51 ± 9.35	17.7 ± 0.6	24.06 ± 9.75*	19.67 ± 3.06	- 39.8	22.3 ± 0.8
50	11.2 ± 0.3*	2.02 ± 1.75	12.2 ± 0.6*	7.25 ± 4.64	39.33 ± 12.70	+ 20.4	16.0 ± 1.0*
Correlation coefficient	-0.78	nc	-0.79	nc	nc	nc	-0.75

Data are means ± SD.

*, values significantly different from control at p < 0.05. Positive values of FNO show that number of organisms was higher in tested groups than within control; negative values mean that the number of individuals was higher in control than in exposed groups; nc, no correlation higher than 0.7 or lower than -0.7 was found.

**Fig 1:**
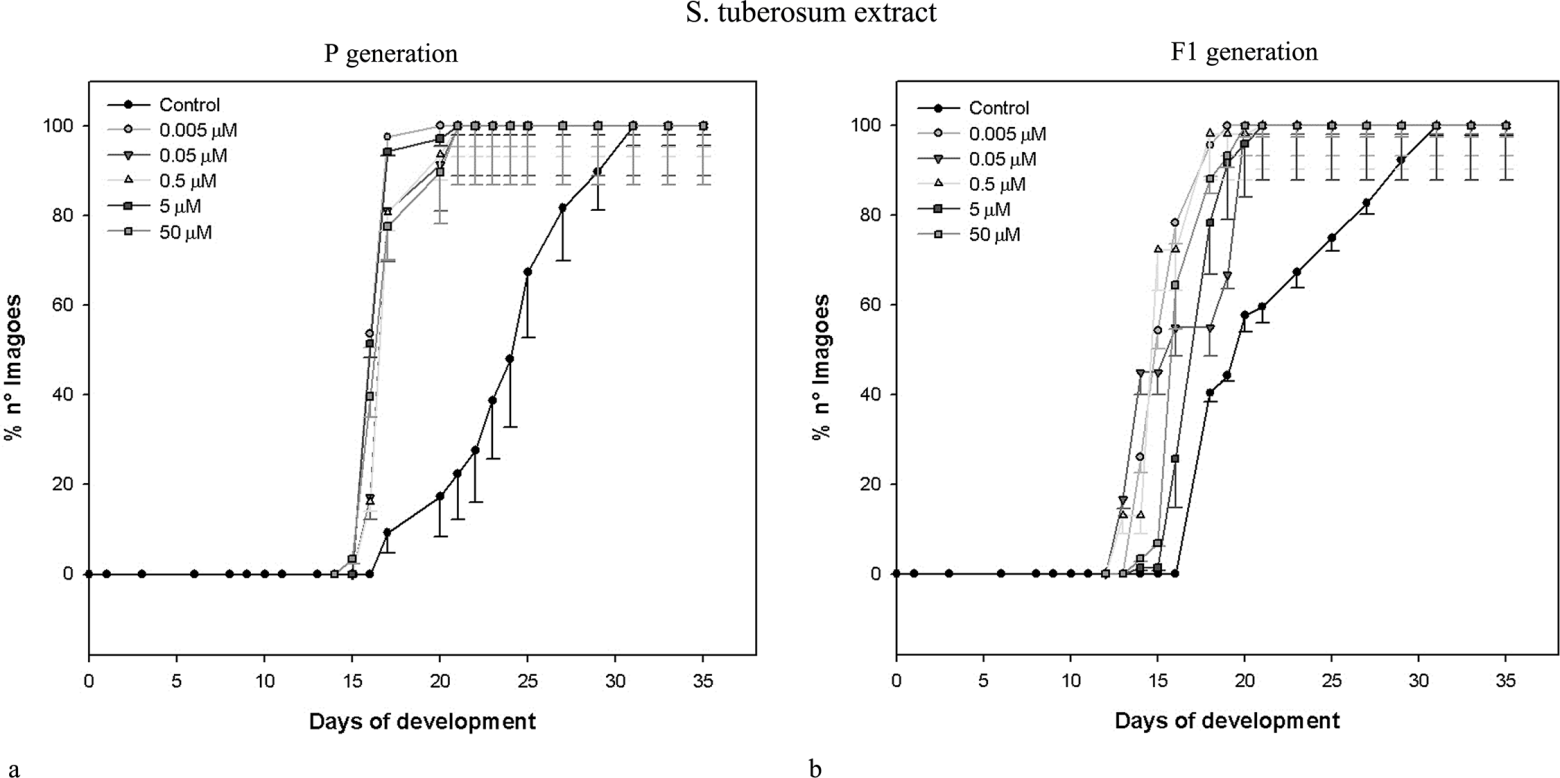
Development of *D*. *melanogaster* fruitflies–effect of *S*. *tuberosum* extract. (a) exposed to the extract; (b) the next (non-exposed, F_1_) generation.

**Fig 2:**
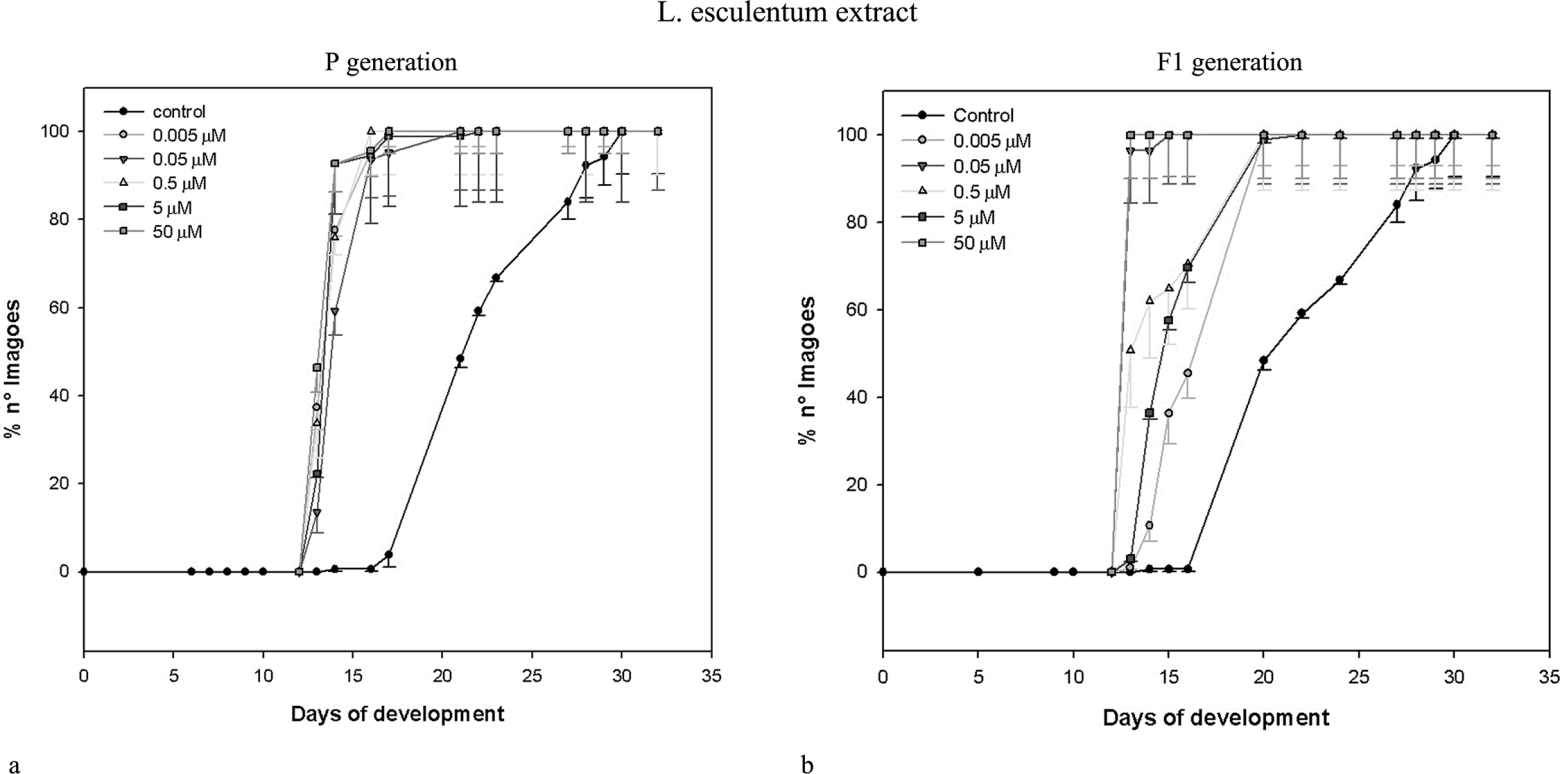
Development of *D*. *melanogaster* fruitflies exposed to *L*. *esculentum* extract. (a) exposed to the extract. (b) the next (non-exposed, F_1_) generation.

**Fig 3:**
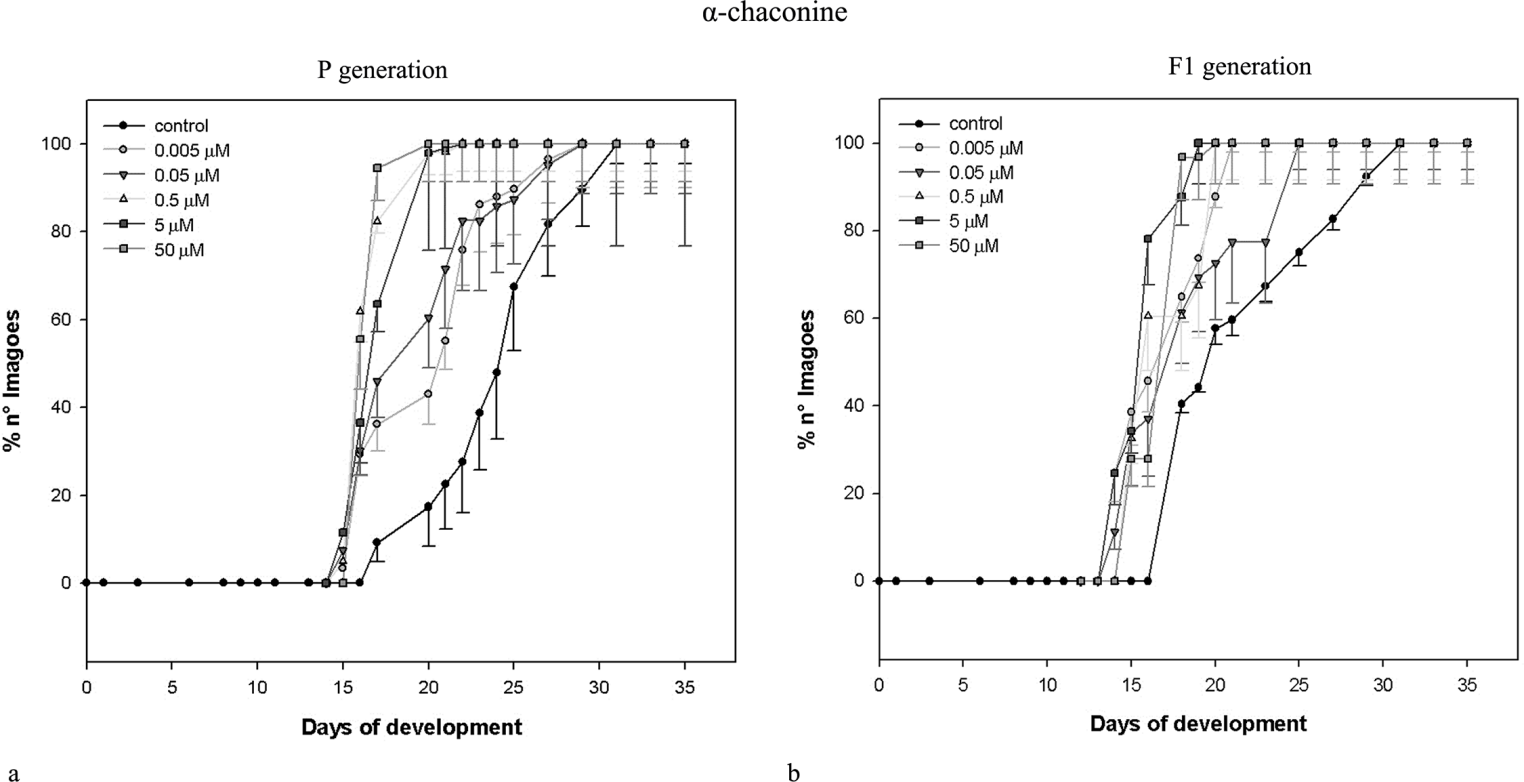
Development of *D*. *melanogaster* fruitflies–effect of α-chaconine. (a) exposed to α-chaconine; (b) the next (non-exposed, F_1_) generation.

**Fig 4:**
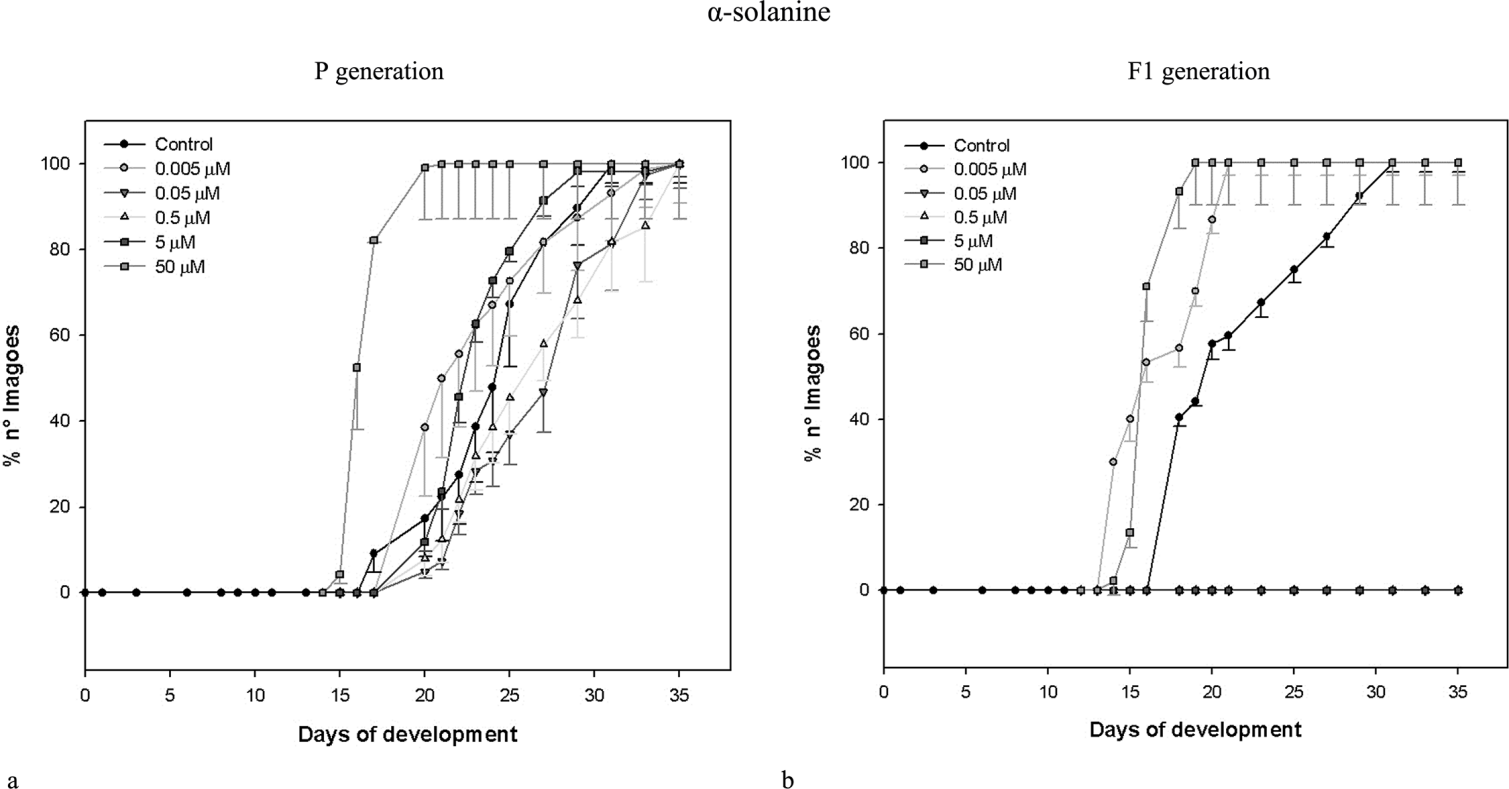
Development of *D*. *melanogaster* fruitflies–effect of α-solanine. (a) exposed to α-solanine; (b) the next (non-exposed, F_1_) generation.

**Fig 5:**
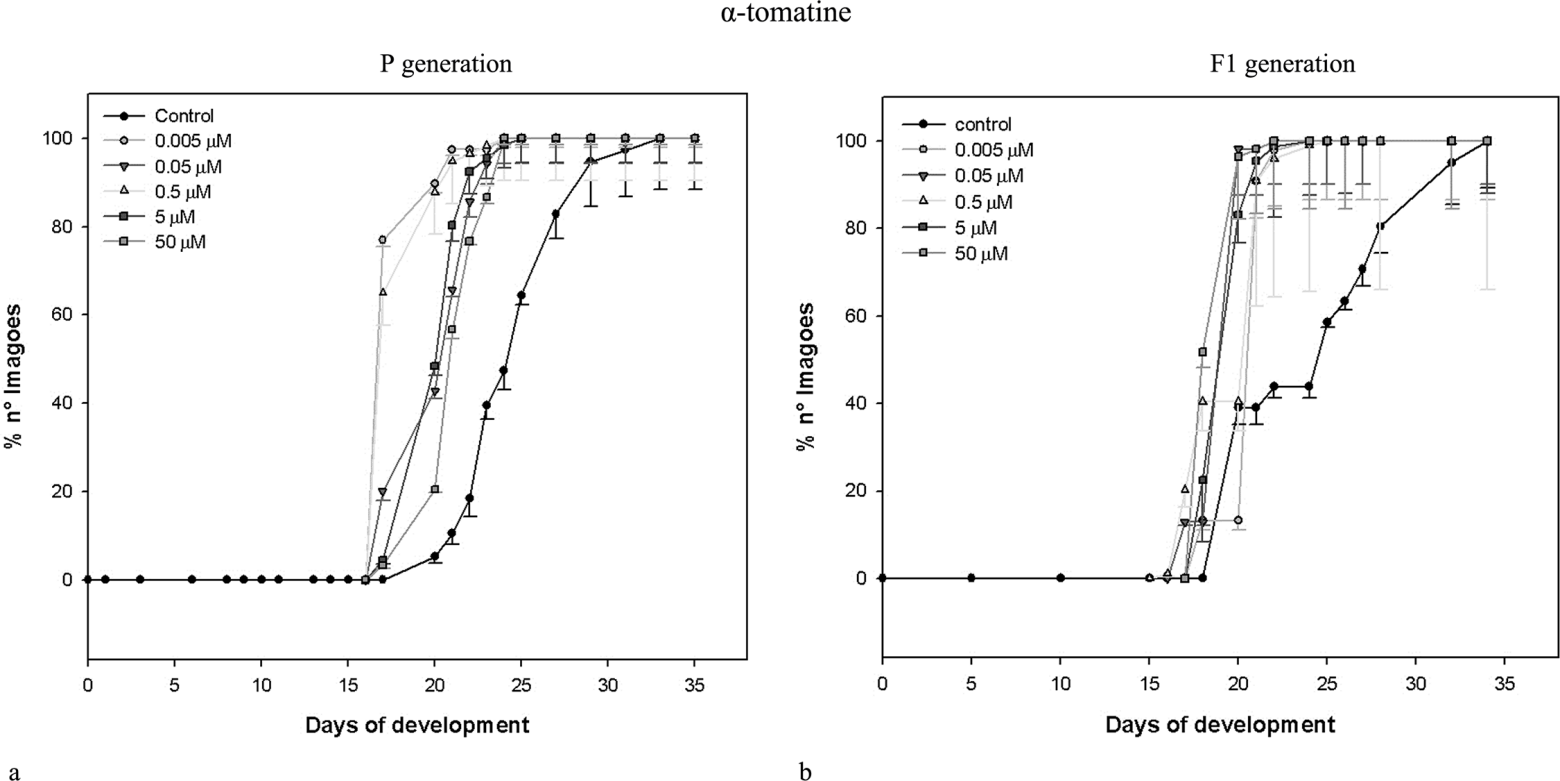
Development of *D*. *melanogaster* fruitflies–effect of α-tomatine. (a) exposed to α-tomatine. (b) the next (non-exposed, F_1_) generation.

Data obtained for the same parameters by the same concentrations of *L*. *esculentum* leaf extract and α-tomatine administered to parental (exposed) generation of *D*. *melanogaster* are shown in Table 2. Similarly to potato extract and its main alkaloids, the effects were non-linear, and correlation coefficients were weak or moderate. Neither extracts nor pure GAs caused drastic mortality of exposed insects. Only the highest concentration of α-tomatine effected in significantly increased mortality of pupae. The most prominent change was the shorter developmental time of insects exposed to both extracts (see also: Figs 1 and 2). All larvae and pupae, and majority of imagoes developed faster, than control ones. Interestingly, potato and tomato extracts seems to cause concentration-independent effects, within tested range of concentrations. They decreased developmental time of c.a. 35%. α-Chaconine had much more significant, concentration-dependent effect (Table 1, Fig 3) than α-solanine, which caused statistically significant effect only when the highest concentration was applied (Table 1, Fig 4). The highest α-solanine concentration (50 μM) caused drastic change in the pattern of the curve of development (Fig 4), while the other tested concentrations followed the control curve. α-Tomatine decreased developmental time but there was no clear concentration-developmental time correlation. The exposure affected rather the range of time of development than the time when the imaginal molt begun: the difference between the first noted imagoes within exposed and control groups was not bigger than two days, while the last imagoes were observed more than 15 days after the first one (Figs 1–5). Also FNO was reduced most significantly in case of insects exposed to extracts, (Tables 1 and 2). α-Chaconine, α-solanine and α-tomatine caused diverse effects depending of concentration. More often we could notice decreased number of organisms. However, the mortality was too low, to calculate lethality on the basis of the probit analysis.

**Table 2:** Effect of *L*. *esculentum* leaf extract and α-tomatine on parental (exposed) generation of *D*. *melanogaster*.

Concentration [μM]	Larvae	Pupae		Imagoes			
	T50 [days}	Mortality [%]	T50 [days]	Malformations [%]	N° final organisms	FNO	T50 [days]
***L*. *esculentum* extract**							
Control	13.2 ± 0.8	1.51 ± 1.42	15.4 ± 0.3	0.00 ± 0.00	52.33 ± 9.61	0.0	21.2 ± 1.0
0.005	7.7 ± 0.0*	7.06 ± 4.36	8.9 ± 0.3*	3.24 ± 2.12	33.67 ± 2.52	- 35.7	13.2 ± 0.3*
0.05	8.5 ± 0.0*	6.64 ± 5.28	9.6 ± 0.3*	8.19 ± 7.09	41.67 ± 13.28	- 20.4	14.0 ± 0.9*
0.5	8.2 ± 0.6*	9.91 ± 8.53	9.7 ± 0.5*	0.00 ± 0.00	27.67 ± 9.87	- 47.1	13.2 ± 0.6*
5	7.8 ± 0.0*	5.26 ± 5.12	9.2 ± 0.3*	3.26 ± 1.93	31.33 ± 16.01	- 40.1	13.3 ± 0.3*
50	7.7 ± 0.3*	6.90 ± 5.94	8.7 ± 0.0*	0.00 ± 0.00	23.00 ± 5.00*	- 56.0	13.0 ± 0.5*
Correlation coefficient	nc	nc	nc	nc	nc	nc	nc
**α-tomatine**							
Control	15.2 ± 0.3	0.00 ± 0.00	19.8 ± 1.7	0.00 ± 0.00	21.33 ± 3.51	0.0	24.2 ± 1.7
0.005	9.5 ± 0.4*	0.00 ± 0.00	13.9 ± 0.5*	0.00 ± 0.00	18.67 ± 2.08	- 12.5	16.5 ± 0.4*
0.05	10.3 ± 0.6*	12.45 ± 11.65	16.4 ± 1.0*	1.51 ± 1.34	11.67 ± 5.69	- 45.3	20.3 ± 1.8*
0.5	10.2 ± 1.6*	4.81 ± 2.79	14.1 ± 0.9*	0.00 ± 0.00	19.00 ± 9.54	- 10.9	16.9 ± 0.4*
5	12.0 ± 0.6*	1.19 ± 1.06	16.3 ± 0.2*	20.56 ± 4.19*	22.67 ± 4.51	+ 6.3	20.0 ± 1.0*
50	12.4 ± 0.9*	19.96 ± 2.28*	16.8 ± 0.2*	15.08 ± 1.38*	14.67 ± 1.16	- 31.2	20.8 ± 0.3*
Correlation coefficient	nc	0.80	nc	nc	nc	nc	nc

Data are means ± SD.

*, values significantly different from control at p < 0.05. Positive values of FNO show that number of organisms was higher in tested groups than within control; negative values mean that the number of individuals was higher in control than in exposed groups; nc, no correlation higher than 0.7 or lower than -0.7 was found.

Both tested extracts affected the second (non-exposed) generation (Tables 3 and 4). Either larval, pupal or imaginal development was significantly faster for both extracts. However, the effect was not concentration-dependent. The extracts affected F_1_ populations much more intensively, than the corresponding standards (α-chaconine and α-tomatine, respectively).

**Table 3:** Developmental parameters of non-exposed (F_1_) generation of *D*. *melanogaster*. Parental generation exposed to *S*. *tuberosum*, α-chaconine or α-solanine.

Concentration (P generation) [μM]	Larvae	Pupae		Imagoes			
	T50 [days]	Mortality [%]	T50 [days]	Malformations [%]	N° final organisms	FNO	T50 [days]
**P exposed to *S*. *tuberosum* extract**							
Control	13.0 ± 0.4	0.00 ± 0.00	14.8 ± 0.2	0.00 ± 0.00	17.33 ± 2.08	0.0	20.2 ± 2.2
0.005	9.4 ± 1.0*	11.28±6.56	9.6 ± 0.7*	11.26 ± 1.55	33.38 ± 17.04	+ 92.6	14.5 ± 0.8*
0.05	7.5 ± 0.5*	0.00 ± 0.00	9.2 ± 0.5*	0.00 ± 0.00	24.71 ± 0.58	+ 42.6	15.3 ± 0.5*
0.5	9.3 ± 0.9*	1.28 ± 1.22	9.5 ± 0.7*	10.86 ± 8.93	20.53 ± 4.51	+ 18.5	14.7 ± 0.2*
5	10.8 ± 1.9	0.00 ± 0.00	11.5 ± 1.2*	2.30 ± 1.98	39.48 ± 6.00*	+ 127.8	16.7 ± 1.0
50	10.2 ± 1.4	3.30 ± 2.92	10.4±0.5*	9.84 ± 3.88	23.76 ± 7.23	+ 37.1	15.4 ± 0.5*
Correlation coefficient	nc	nc	nc	nc	nc	nc	nc
**P exposed to α-chaconine**							
Control	13.0 ± 0.4	0.00 ± 0.00	14.8 ± 0.2	0.00 ± 0.00	17.33 ± 2.08	0.0	20.2 ± 2.2
0.005	11.5 ± 0.5	3.50 ± 2.12	9.5 ± 0.5*	16.34 ± 13.20	8.87 ± 2.00	- 48.8	14.5 ± 0.5*
0.05	10.0 ± 1.4	6.02 ± 3.27	10.0 ± 0.4*	21.86 ± 21.18	31.28 ± 2.00	+ 80.5	17.4 ± 3.0
0.5	10.0 ± 1.6	22.08 ± 17.92	14.9 ± 3.4	0.00 ± 0.00	8.87 ± 8.50	- 48.8	14.8 ± 1.4*
5	11.2 ± 1.1	2.22 ± 1.85	10.5 ± 0.2*	14.60 ± 14.53	63.81 ± 9.29*	+ 268.2	16.1 ± 1.7
50	11.2 ± 3.0	0.00 ± 0.00	12.4 ± 0.2	26.74 ± 10.43*	57.05 ± 9.29*	+ 229.2	16.7 ± 0.5
Correlation coefficient	nc	nc	nc	nc	nc	nc	nc
**P exposed to α-solanine**							
Control	13.0 ± 0.4	0.00 ± 0.00	14.8 ± 0.2	0.00 ± 0.00	17.33 ± 2.08	0	20.2 ± 2.2
0.005	10.3 ± 0.5*	0.00 ± 0.00	10.7 ± 0.5*	9.70 ± 5.78	10.00 ± 3.00	- 42.3	15.4 ± 0.5*
0.05	nd	nd	nd	nd	nd	nd	nd
0.5	nd	nd	nd	nd	nd	nd	nd
5	nd	nd	nd	nd	nd	nd	nd
50	10.2 ± 0.7*	16.67 ± 15.87	9.8 ± 1.7*	1.85 ± 1.61	15.00 ± 9.85	- 13.4	15.0 ± 1.4*
Correlation coefficient	nc	1	nc	nc	nc	nc	nc

Data are means ± SD.

*, values significantly different from control at p < 0.05; nd, no data due to death of all insects. Positive values of FNO show that number of organisms was higher in tested groups than within control; negative values mean that the number of individuals was higher in control than in exposed groups; nc, no correlation higher than 0.7 or lower than -0.7 was found.

**Table 4:** Developmental parameters of non-exposed (F_1_) generation of *D*. *melanogaster*. Parental generation exposed to *L*. *esculentum* or α-tomatine.

Concentration (P generation) [μM]	Larvae	Pupae		Imagoes			
	T50 [days]	Mortality [%]	T50 [days]	Malformations [%]	N° final organisms	FNO	T50 [days]
**P exposed to *L*. *esculentum* extract**							
Control	12.5 ± 1.0	0.00 ± 0.00	15.0 ± 0.6	0.00 ± 0.00	18.00 ± 3.86	0.0	20.6 ± 1.5
0.005	10.6 ± 2.8	0.00 ± 0.00	11.7 ± 1.2	9.45 ± 6.98	33.00 ± 5.00	+ 83.3	16.4 ± 1.2*
0.05	6.9 ± 1.2*	4.00 ± 3.93	7.5 ± 1.2*	0.00 ± 0.00	14.00 ± 8.00	- 22.2	12.5 ± 0.0*
0.5	6.9 ± 1.9*	3.03 ± 2.85	8.3 ± 2.4*	0.95 ± 0.65	23.67 ± 12.66	+ 31.5	13.0 ± 2.6*
5	8.5 ± 0.0*	1.96 ± 1.40	11.3 ± 1.0*	5.34 ± 4.64	16.67 ± 0.58	- 7.4	14.7 ± 3.3*
50	6.8 ± 0.6*	5.33 ± 5.24	7.6 ± 0.6*	1.59 ± 0.95	14.00 ± 7.00	- 22.2	12.5 ± 0.0*
Correlation coefficient	nc	nc	nc	nc	nc	nc	nc
**P exposed to α-tomatine**							
Control	15.3 ± 2.3	0.00 ± 0.00	18.0 ± 4.2	0.00 ± 0.00	17.67 ± 8.02	0.0	24.5 ± 3.3
0.005	13.8 ± 0.0	0.99 ± 0.86	13.8 ± 0.6	1.64 ± 1.32	71.50 ± 9.50*	+ 304.6	20.5 ± 2.0
0.05	12.5 ± 0.3*	0.95 ± 0.65	12.9 ± 0.6	11.11 ± 10.24	27.00 ± 7.00	+ 52.8	18.9 ± 1.7*
0.5	13.3 ± 2.4	17.12 ± 16.44	14.0 ± 0.9	21.57 ± 2.98	33.00 ± 21.50	+ 86.8	20.2 ± 1.8
5	13.2 ± 0.8	1.36 ± 1.19	14.0 ± 1.1	0.00 ± 0.00	44.00 ± 9.00	+ 149.0	19.0 ± 2.0
50	13.7 ± 0.5	9.80 ± 6.74	14.0 ± 1.9	8.90 ± 6.45	28.00 ± 11.00	+ 58.5	18.0 ± 0.8*
Correlation coefficient	nc	nc	nc	nc	nc	nc	nc

Data are means ± SD.

*, values significantly different from control at p < 0.05. Positive values of FNO show that number of organisms was higher in tested groups than within control; negative values mean that the number of individuals was higher in control than in exposed groups; nc, no correlation higher than 0.7 or lower than -0.7 was found.

Interestingly, we did not obtained larvae from three α-solanine F_1_ groups: 0.05, 0.5 and 5 μM (Table 3). The insects were just noted in the lowest and the highest concentration. In contrast to exposed generation, the majority of tested substances (all concentrations of potato extract, 0.05, 5 and 50 μM α-chaconine, 0.005 and 0.05 μM tomato extract and all α-tomatine concentrations) increased number of organisms. On the other hand, the number of imagoes was lower for 0.005 and 0.5 μM α-chaconine, both α-solanine concentrations, 0.05, 5 and 50 μM tomato extracts, compared to control.

The dynamics of F_1_ *D*. *melanogaster* development resembled that of the exposed generation, i.e. if the insects exposed to tested substances developed faster than the control ones, their offspring developed faster, too (Figs 1B–5B), although the effect was weakened. Again, the difference between appearance of first insects was not very significant, but exposed populations reached 100% of imagoes much faster, than the control ones. The difference is the most clear in case of *L*. *esculentum* extract and α-tomatine.

**Fig 6:**
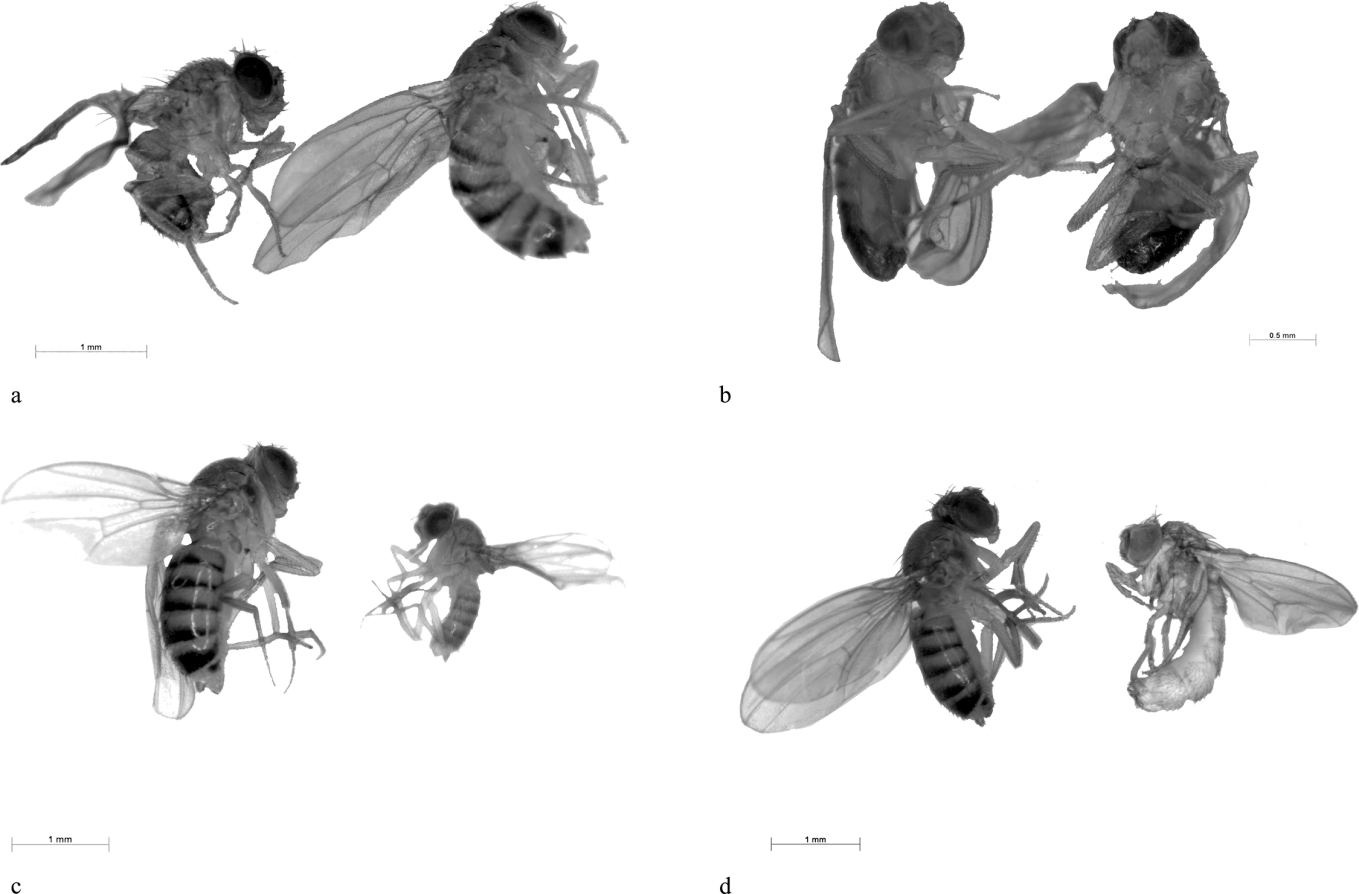
Examples of the most frequent malformations and anomalies caused by exposition to tested extracts and metabolites in comparison with typical imagoes. (a) deformed wings (left, 0.5 μM α-solanine), (b) smaller black abdominal zone (black arrow, left, 50 μM potato extract), (c) smaller body size (right, 5 μM potato leaf extract), (d) deformed abdomen (right, 50 μM potato extract).

The imagoes of both tested generations showed several types of malformations and anomalies, such as: smaller body size, deformed wings and abdomens, smaller abdominal black zone (Fig 6). Potato extracts and α-solanine (P) displayed the highest percentage of total malformed imagoes (Tables 1 and 3). The imagoes of both generations that were reared on culture medium (control) did not show any of the above mentioned malformations. Interestingly, α-chaconine had higher effect on development (T50) than α-solanine, but α-solanine caused more frequent malformations than α-chaconine. This also supports the hypothesis that GAs in crude extracts may have a synergic activity allowing them to protect plants against insects better, than single, pure substances. Moreover, such situation is not an exception in nature.

## Discussion

Pests may slowly reveal resistance against new natural substances found in their natural habitat. However plants constantly produce secondary metabolites (alkaloids, glycoalkaloids, terpenoids, organic acids or alcohols) which are the most important lines of plant defense against various insects [21]. The range of cellular targets for these substances is very wide and covers metabolic pathways, macromolecules (e.g., proteins or nucleic acids) and organelles (e.g., biological membranes or nuclei). Thus they are regarded as promising sources of plant-protecting substances [21]. Therefore, GAs may be an important tool in plant protection. Even if acute toxicity is not high, substances like pesticides and secondary metabolites may weaken exposed population, change its developmental time, postpone molts or inhibit reproduction [29, 30] In consequence, insects may reach next developmental stage in the inappropriate time, can be less resistant to diseases or easier caught by predators. On the basis of the comparison of the results obtained for single metabolites and extracts, we think that mixtures of GAs composing the tested extracts could act synergistically increasing their toxic effects. This hypothesis is supported by several studies [16]. Potato extract showed higher toxicity than single GAs on adults of the rust red flour beetle *Tribolium castaneum* and the rice weevil *Sitophilus oryzae*. The extract exhibited considerable acute and residual toxicity against these insects in a dose-dependent manner and was more toxic than single purified phytochemicals indicating their synergistic interaction [20]. Our results show, that both main alkaloids reveal different action on fruitflies. The extract and α-chaconine caused concentration-dependant mortality of P-larve, whereas α-solanine decreased duration of development, in a concentration-dependant manner. However, this effect was weaker, than in case of α-chaconine and crude extract, which revealed the highest effect. That proves, that both alkaloids act synergistically. The phenomenon of α-chaconine causing higher effect on developmental time (T_50_) than α-solanine, and more frequent malformations observed in insects exposed to α-solanine than α-chaconine also supports the hypothesis that GAs in crude extracts may have a synergic activity allowing them to protect plants against insects better, than single, pure substances. Such situation is not an exception in nature [31–33]. Synergistic action of various substances can cause stronger, wider range of toxic effects within herbivores and limit concentration of substances produced by plants. Similarly to our research, potato extract and its major GAs displayed considerable contact toxic effects on khapra beetle *Trogoderma granarium* when a topical application technique was used. Also in this case extract was more toxic than single phytochemicals. Potato GAs reduced the growth rate, food consumption rate and food utilization by adults of insects [34]. The toxicity of single pure GAs was demonstrated. In particular, α-tomatine caused the greatest mortality of potato leafhopper, *Empoasca fabae* compared with other GAs such as α-solanine, α-chaconine, leptine I and II, solasonine and solamargine [11]. α-Tomatine was also highly toxic on deposited eggs of the diamondback moth (*Plutella xylostella*) that attacks cruciferous vegetables such as cabbage [8]. Hatching of eggs decreased from 90 to 20% after treatment with a 0.1% α-tomatine solution. Larval growth of red flour beetles was inhibited on artificial diets containing 1 μmol/g of solamargine, solasonine and α-tomatine, while only the last GA showed marked inhibitory activity on tobacco hornworm *Manduca sexta* [13].

Non-linear effects of Solanaceae alkaloids were also reported. α-Solanine was described as an agent that stimulates the growth of human fibroblasts, while its higher higher concentrations inhibited cell divisions [35]. The authors suggested, that the alkaloid, due to its structure, mimics steroid sex hormones. Similar situation may take place in case of insects, where steroid hormones play crucial role in development and molting. Observed decrease of the body size is in tune with previous finding reporting reduced body size of imagines of various *Drosophila* species in response to exposure to alkaloids present in plants where they had developed as larvae [36, 37]. Also developmental alteations were reported in case of *D*. *melanogaster* exposed to alkaloids, with remarkably shorter time of development [38].

The number of F_1_ (non-exposed) organisms was higher than control for both Solanaceae plants’ extracts and single GAs. Probably, the higher number of adult insects was due to directional selection that took place during exposure. The final number of organisms was much lower in exposed groups than in control. The weakest insects did not survive or had lower vigour. Therefore, only stronger individuals reproduced, what effected in the increased number of laid eggs and stronger offspring. Control insects were still under stabilizing selective pressure. Similar effect was observed in case of *Pimpla turinellae* exposure to malathion, an organophosphate insecticide. It increased the number of eggs laid by adult females [39]. *Spodoptera exigua* moths exposed to another organophosphate insecticide fenitrothion caused higher resistance to this pesticide, after three generations [40]. Insects in F_1_ generation revealed some malformations but their number was statistically insignificant. Perhaps, the metabolites possess also some genotoxic effect, but this must be carefully checked. Next, since α-solanine has lower effect on development, but it causes more malformations, the individuals that reached imaginal stage earlier could possess more malformations than those that reached imaginal stage later. The effect of tested samples on the growth rate of fruit flies is very interesting. In case of experiment with culture medium containing silver nanoparticles, adaptation was observed in the eight generation based, similarly to our data, on the shortening of the development time. *Drosophila* larvae "minimized" the consumption time in order to diminish the adverse effects and the fecundity of flies consequently increased [25]. The presence of the extracts and their respective main metabolites in food resulted in faster growth. α-Solanine showed significant effect on the rate of development of parent imagoes with respect to control only at highest tested concentration. At intermediate concentrations, the growth of parent fruitflies did not significantly varied but no third instar larva or pupa of F_1_ generation was observed in vials. Probably, larvae didn’t hatch or organisms died during their first two larval stages.

It can be noted that moderate concentrations of α-solanine in P generation caused higher amount of malformations, than the lowest and the highest one. Additionally, 0.05 and 5μM concentrations caused higher mortality within exposed generation, than 0.05 and 50μM concentrations. Together, these effects lead to lower vigor or even extinction of the next generation. On the other hand, the lowest concentration of α-solanine could be undetectable by parent insects. In consequence they ingested a greater quantity of toxins and their detoxifying mechanisms were not significantly activated, so α-solanine produced toxicity on organisms belonging to F_1_ generation. Some studies showed an opposite effect of GAs on growth rate of insects. Rearing of Colorado potato beetle (*Leptinotarsa decemlineata*) on a synthetic diet supplemented with increasing concentrations of α-tomatine resulted in delayed development [8]. α-Tomatine (25-200 μM) decreased larval survival, lowered pupal weight, an extended pupation period and the time of imaginal molt of Mediterranean fruitfly *Ceratitis capitata* [8, 41]. Probably, *L*. *decemlineata* and *C*. *capitata* development was delayed because they slowly metabolized the GAs. Besides, both species are widely distributed, are able to tolerate various stress factors. Therefore, their detoxifying mechanisms may be better adjusted to GAs. In consequence, these insects can tolerate the toxic compounds better than *D*. *melanogaster*, which is very sensitive. Both strategies may be successful and result in increased survival. For example, the organophosphorus pesticides dichlorvos and chlorpyrifos increased the population growth rate of fresh water rotifer *Brachionus calyciflorus* [42].

Even if further experiments on lethal and sublethal insecticidal activity on various life stages and strains of *D*. *melanogaster* must be carried out, we would like to emphasize, that although the lethality of tested substances was usually statistically not significant, Solanaceae extracts caused various sublethal effects on development and reproduction of two consecutive generations of *D*. *melanogaster* wild type. The activity of extracts is often more significant, than in case of pure GAs. Moreover, due to selective pressure, GAs may affect populations in long-term period. Therefore, they may play important role in crop protection, both in organic agriculture, where they can increase availability of pests to their predators and in pesticide-treated fields, where plant extracts may act synergistically with chemical insecticides, helping to significantly reduce their use, as required by the most current agricultural policies and consumers. These data suggest, that Solanaceae can be a source of economically and ecologically important substances, that can be used in protection of natural and agricultural habitats.
